# Multiple inflammatory cytokines correlate with the vestibular and oculomotor dysfunction in Fabry disease: a prospective, longitudinal study

**DOI:** 10.3389/fimmu.2026.1658002

**Published:** 2026-04-17

**Authors:** Yinglin Leng, Yujing Yuan, Yawen Zhao, Xia Wang, Jing Ma, Xinyu Zhang, Jingyi Huang, Hongjun Hao, Guiping Zhao, Wei Zhang

**Affiliations:** 1 Department of Neurology, Peking University First Hospital, Beijing, China; 2 Department of Neuroimmunity, Peking University First Hospital, Beijing, China; 3 Beijing Key Laboratory of Neurovascular Diseases, Beijing, China; 4 Rare Disease Medical Center, Peking University First Hospital, Beijing, China

**Keywords:** Fabry disease, inflammatory cytokines, oculomotor, vestibular dysfunction, videonystagmography

## Abstract

**Objectives:**

Vestibular and oculomotor abnormalities have been widely identified in Fabry disease (FD), with inflammation potentially playing an important role. We aim to investigate the expression of inflammatory cytokines (ICs) in FD patients and their relationship with the vestibular/oculomotor dysfunctions.

**Methods:**

This prospective observational study enrolled 40 FD patients. All 40 patients underwent the visuo-oculomotor examination, and 22 of them received the vestibulo-oculomotor examination. Plasma concentrations of 14 ICs were detected, including interferon-γ, interleukin (IL)-1β, IL-2, IL-4, IL-5, IL-6, IL-8, IL-10, IL-12p70, IL-17A, IL-17F, IL-22, tumor necrosis factor (TNF)-α, and TNF-β. Statistical analyses were made between different subgroups of patients.

**Results:**

(1) In the visuo-oculomotor examination, TNF-β was significantly higher in patients with prolonged saccade latency (1.61 ± 0.38 VS 1.14 ± 0.39, p=0.001) and hypometria (1.46 ± 0.39 VS 1.18 ± 0.48, p=0.043) than in patients without those abnormalities. The average saccade latency was positively correlated with the level of TNF-β (r=0.378, p=0.021), while the average saccadic accuracy was negatively correlated with the level of TNF-β (r=-0.333, p=0.044). IL-12p70 was significantly elevated in patients with defective pursuit compared to patients with normal pursuit (1.63 ± 0.20 VS 1.21 ± 0.54, p=0.040). (2) In the patients with vestibulo-oculomotor dysfunction, the plasma levels of IL-2 (3.40 ± 1.00 VS 2.13 ± 0.91, p=0.007), IL-17A (6.42 ± 3.59 VS 3.05 ± 2.13, p=0.021) and TNF-β (1.55 ± 0.41 VS 1.21 ± 0.37, p=0.030) were significantly elevated compared to the patients with normal vestibulo-oculomotor function.

**Conclusions:**

Inflammation-mediated pathological mechanism, especially TNF-β-related pathways, is associated to both central and peripheral vestibular dysfunction in FD patients.

## 1 Introduction

Fabry disease (FD) is a rare X-linked inherited disorder caused by mutations in the GLA gene, leading to α-galactosidase A (α-GalA) deficiency (1). The subsequent accumulation of globotriaosylceramide (Gb3) in tissues damages multiple organs, including brain, heart, kidney, and eyes (2). Vertigo and dizziness, resulting from the involvement of vestibular system, are among the main symptoms of FD (3, 4). Several studies, including our previous work, suggest that the central oculomotor abnormalities (e.g. smooth pursuit impairment, saccadic abnormalities) and peripheral vestibular dysfunction, which are more prominent than in age-matched healthy controls, may be related to the substrate deposition in small blood vessels of the brain and inner ear (5–9). The FD patients with defective saccades demonstrate higher small-vessel disease score (SVDS) and elevated plasma Gb3 levels (9).

In recent years, the role of inflammation induced by substrate deposition has been established as an important mechanism of organ damage in FD, particularly affecting cardiac and renal systems (10). Our previous studies demonstrated elevated plasma levels of multiple inflammatory cytokines (ICs) in FD patients, including interferon (IFN)-γ, interleukin (IL)-1β, IL-5, IL-8, and tumor necrosis factor (TNF)-β, which showed positive correlations with disease burden (as measured by the Mainz Severity Score Index [MSSI]) and markers of cardiac and renal dysfunction (11). It can be speculated that inflammatory mechanisms may also contribute to the pathogenesis of vestibular damage in FD. Therefore, we designed this study to investigate whether elevated ICs are associated with vestibular and oculomotor abnormalities in patients with Fabry disease.

## 2 Methods

### 2.1 Patient enrollment

**Figure 1:**
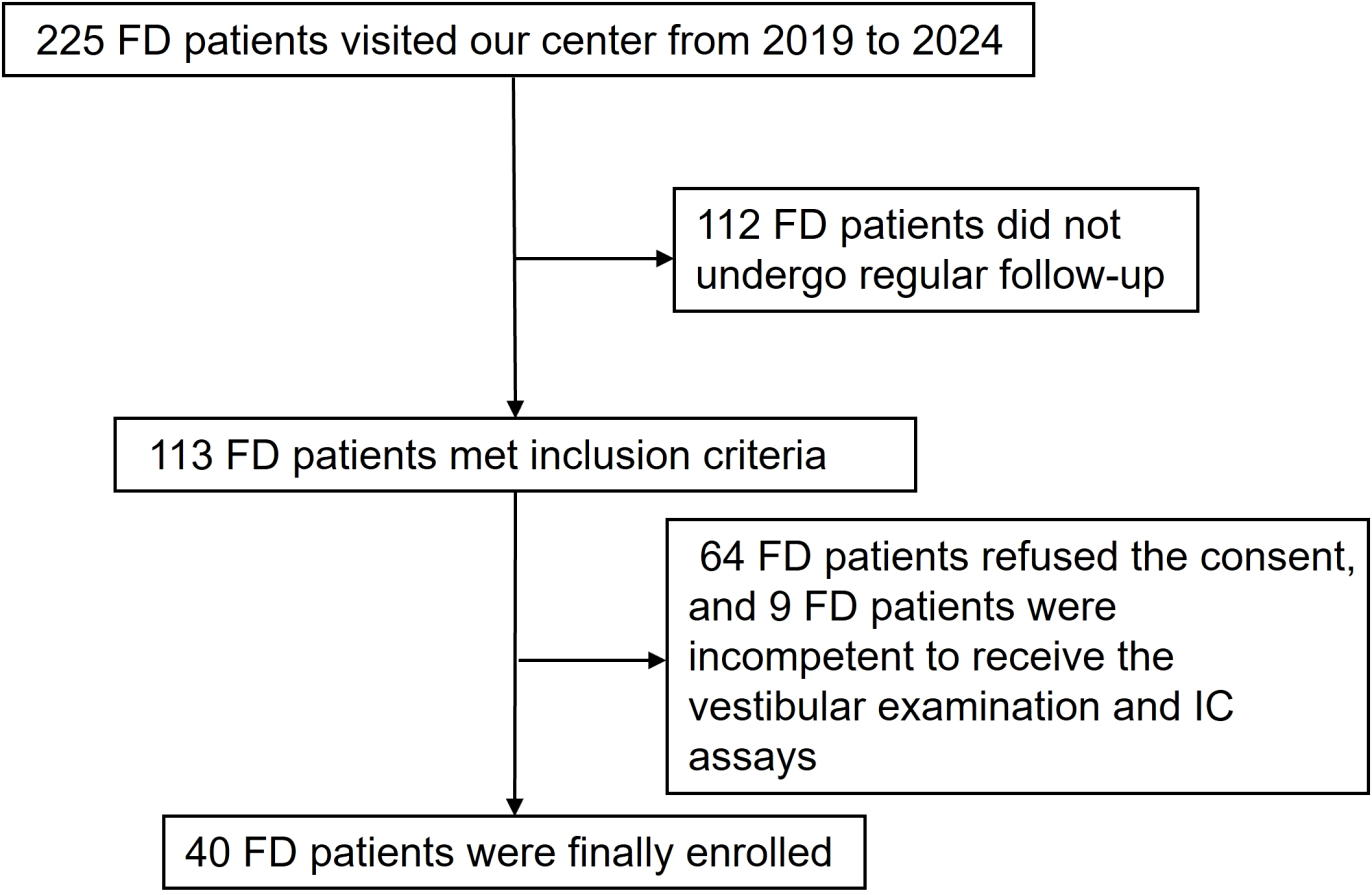
Flowchart of patient recruitment. Forty participants were selected from 225 consecutive FD patients at our center based on defined inclusion and exclusion criteria.

This prospective observational study enrolled 40 patients with FD (age range: 13–64 years) selected from 225 FD patients who visited Peking University First Hospital between Jan 2019 to April 2024 (**Figure 1**). Inclusion criteria: (1) diagnosis confirmed by typical clinical manifestations, family history and laboratory findings (including GLA gene, α-Gal A activity, and plasma Lyso-Gb3) according to Chinese consensus in 2021 (12), (2) maintaining regular follow-up. Exclusion criteria: (1) refusal to participate, (2) incapability to complete vestibular examinations due to severe comorbidities (such as severe subjective vertigo/dizziness assessed by clinical interview, severe visual/hearing/motor impairments, acute coronary syndrome, etc.) or cognitive impairment, (3) concurrent conditions potentially affecting inflammatory status, such as infectious disease, tumors, autoimmune disease, immunomodulatory therapy, etc. Written informed consent for participation and publication were obtained from the patients or their guardians during follow-up visits. No control subjects were recruited as comparisons were only internal between dysfunction/non-dysfunction subgroups.

### 2.2 Vestibular examinations

All vestibular examinations were performed using standardized equipment and followed a fixed protocol by two trained and experienced examiners (WX and MJ) to ensure inter−rater consistency and procedural standardization.

#### 2.2.1 Visuo-oculomotor examination

The visuo-oculomotor examination included recording of oculomotor movements by a computer-based videonystagmography (VNG) system (Bao Runtong Research Ltd. China). The participant was first asked to look forward in a dark room without any target and eye movements were recorded for 60s to identify spontaneous nystagmus and saccadic intrusions. Then several visuo-oculomotor tasks were performed as follows (9, 13).

##### 2.2.1.1 Gaze-holding test

1. Procedure: the participant was asked to fix their gaze on a central target, and then on eccentric targets of ±15° horizontally, 10 s in each position.
2. Recording: nystagmus beating toward the direction of the target during the fixation was recorded as gaze-evoked nystagmus (GEN).

##### 2.2.1.2 Reflexive saccade test

1. Procedure: the participant was instructed to fixate on the central spot (0°). The primary target extinguished simultaneously with the appearance of a peripheral target. Timing (1.0–1.5 s) and position of the target (2 times of ±5°, ± 10°, ± 15°, ± 20°, ± 25°, and ±30° horizontally) appeared randomly on the screen. The participant was instructed to visually track the target as rapidly as possible.
2. Recording: the latency (the interval between target presentation and the start of the saccade), peak velocity, and accuracy/gain (saccade amplitude/target amplitude) of each saccade were measured by the computer. Abnormal saccades with prolonged latency (>250ms), slow peak velocity (below different reference value of each angle), hypometria (decreased gain <0.7), hypermetria (increased gain >1.15) and dysmetria (gain <0.7 or >1.15) were qualitatively recorded (14).

##### 2.2.1.3 Smooth pursuit (SPM) test

1. Procedure: the participant was required to visually pursuit a sinusoidally moving target smoothly. The target moved in the horizontal direction with an amplitude of ± 15° at a frequency of 0.2 Hz.
2. Recording: the gain (eye velocity/target velocity) for the left and the right directions were calculated separately by computer. Defective SPM were defined as pursuit gain decreased below 0.6 or saccadic wave appeared upon sinusoidally trajectory (saccadic pursuit).

##### 2.2.1.4 Optokinetic test

1. Procedure: the participant was asked to visually track a series of targets continuously moving at a constant speed in the whole vision field as rapidly as possible, and the optokinetic nystagmus (OKN) was induced.
2. Recording: the gain (the slow phase velocity of OKN/target velocity) was calculated by the computer, and defective OKN was judged with a gain < 0.6.

#### 2.2.2 Vestibulo-oculomotor examination

The vestibulo-oculomotor examination included the caloric test and the video head-impulse test (vHIT).

##### 2.2.2.1 Caloric test

1. Procedure: 250 ml of 30 °C-cold and 44 °C-warm water was irrigated unilaterally into each ear during 30 s, then eye movement responses were recorded for 90 s by the VNG system.
2. Recording: the slow phase velocity (SPV) of the nystagmus induced by cold and warm water in each ear, reflecting the function of vestibulo-ocular reflex (VOR) to low-frequency stimulation, was calculated by the computer, recorded as LC, LW, RC and RW. The canal paresis (CP, or dissymmetry ratio) was calculated as follows:

$$CP=\frac{\left(RW+RC)-(LW+LC\right)}{\text{R}W+RC+LW+LC}\times 100\%$$

Unilateral weakness (UW) of the horizontal semicircular canal (SC) was defined as the summation of SPV in one ear (i.e., LC+LW, or RC+RW) <12°/s, or |CP|>30%.

Bilateral weakness (BW) was defined as both LC+LW and RC+RW<12°/s.

##### 2.2.2.2 Video head-impulse test

1. Procedure: rotational head thrusts were applied approximately along the planes of the horizontal, left anterior and right posterior (LARP), and right anterior and left posterior (RALP) SCs by an investigator standing behind the subject.
2. Recording: three-dimensional eye and head movements were measured by the ICS impulse vestibular testing system (Natus Medical Incorporated). If the gain (eye velocity/head velocity, reflecting the function of VOR in the high-frequency range) in any thrust direction was < 0.7 or compensatory saccades were recorded, the function of the SC in that direction was interpreted as abnormal.

### 2.3 IC assay

Peripheral whole blood was collected in ethylenediaminetetraacetic acid tubes and immediately centrifuged at 1240×g for 5 min at 4 °C. Plasma was isolated and frozen at −80 °C before further processing. Sample preparation and detection of cytokines—namely, IFN-γ, IL-1β, IL-2, IL-4, IL-5, IL-6, IL-8, IL-10, IL-12P70, IL-17A, IL-17F, IL-22, TNF-α, and TNF-β—were performed using a sandwich enzyme-linked immunosorbent assay kit (914002, QuantoBio, Tianjin, China) following the manufacturer’s instructions. The cytokine concentrations of each sample were detected using a flow cytometer (BeamCyte-1026M, Jiangsu, China) and analyzed using CYTOSYS 2.0 software (Changzhou Bidako Biotechnology Co., Ltd., Changzhou, China) (11).

### 2.4 Statistical analysis

IBM SPSS Statistics for Windows, version 26.0 (IBM Corp., Armonk, NY, USA) was used for all analyses. ICs expression was compared between subgroups with different vestibular examination changes (i.e. with/without visuo-oculomotor or vestibulo-oculomotor deficiency, slow saccades, prolonged saccadic latency, hypometria, hypermetria, defective saccades, defective SPM, and defective OKN). The Shapiro–Wilk test was applied to determine variable distributions. Data with a normal distribution was given as the mean ± standard deviation (SD). Data with a non-normal distribution was given as the median (interquartile range, IQR). The Mann–Whitney U test was used to compare differences between subgroups. Pearson’s methods (for data with a normal distribution) or Spearman’s methods (for data with a non-normal distribution) were used for correlation analyses between IC levels and the parameters of vestibular examinations (i.e. saccadic latency, saccadic accuracy, saccadic velocity, and gain of SPM). A p-value of <0.05 was considered significant.

## 3 Results

### 3.1 Patients characteristics

This study included 40 FD patients (23 males, 17 females) from 33 unrelated families, with the mean (± SD) age of 37.25 (± 13.67) years at sample collection. One female of the patients was asymptomatic and identified through family screening. The remaining 39 symptomatic patients showed a median (IQR) onset age of 8.00 (3.00) and mean (± SD) disease duration of 25.07 (± 14.08) years. Genetic analysis of all 33 families identified GLA variants, with exon mutations in 32 families (17 missense, 1 in-frame, 1 splice, 2 insertion/deletion, 9 nonsense, and 2 frameshift mutations) and an intron insertion in 1 family (see **Supplementary Table 1** in **Supplementary Material**).

### 3.2 Visuo-oculomotor examination changes and the relationship with IC expression

The visuo-oculomotor examination was conducted on all 40 patients, with 34 (85.0%) of them showed abnormalities (**Table 1**).

**Table 1:** Visuo-oculomotor examination findings.

Examinations	Parameters	Patient characteristics (n=40)	Reference range
Spontaneous nystagmus		8(20.0%)	
Gaze-evoked nystagmus		1(2.5%)	
Reflexive saccade test	Abnormality	31(77.5%)	
	Slow saccade	15(37.5%)	
	Increased latency	17(42.5%)	
	Hypometria	23(57.5%)	
	Hypermetria	15(37.5%)	
	Average velocity (5°), °/s	122.2 ± 18.7	>75
	Average velocity (10°), °/s	197.8 ± 28.1	>135
	Average velocity (15°), °/s	254.4 ± 44.6	>195
	Average velocity (20°), °/s	302.0 ± 44.9	>246
	Average velocity (25°), °/s	375.6 ± 89.4	>285
	Average velocity (30°), °/s	394.4 ± 60.5	>320
	Average latency, ms	194.1 ± 54.5	<250
	Average accuracy	0.91 ± 0.07	0.7~1.15
Saccadic intrusion		16(40.0%)	
Smooth pursuit test	Abnormality	9(22.5%)	
	Saccadic pursuit	9(22.5%)	
	Decreased SPM gain	5(12.5%)	
	Average SPM gain	0.76(0.13)	≥.76
Impaired optokinetic nystagmus		5(12.5%)	
Visuo-oculomotor Abnormality		34(85.0%)	

Data are mean ± SD, N or median(IQR) values. SPM, smooth pursuit movement.

#### 3.2.1 Abnormalities in reflexive saccade test

Thirty-one (77.5%) patients showed impaired saccades, including slow saccades (15, 37.5%), increased saccade latency (17,42.5%), hypometria (23, 57.5%) and hypermetria (15, 37.5%). Saccadic intrusions appeared in 16 (40.0%) patients, manifesting as square-wave jerks or square-wave pulses.

**Figure 2:**
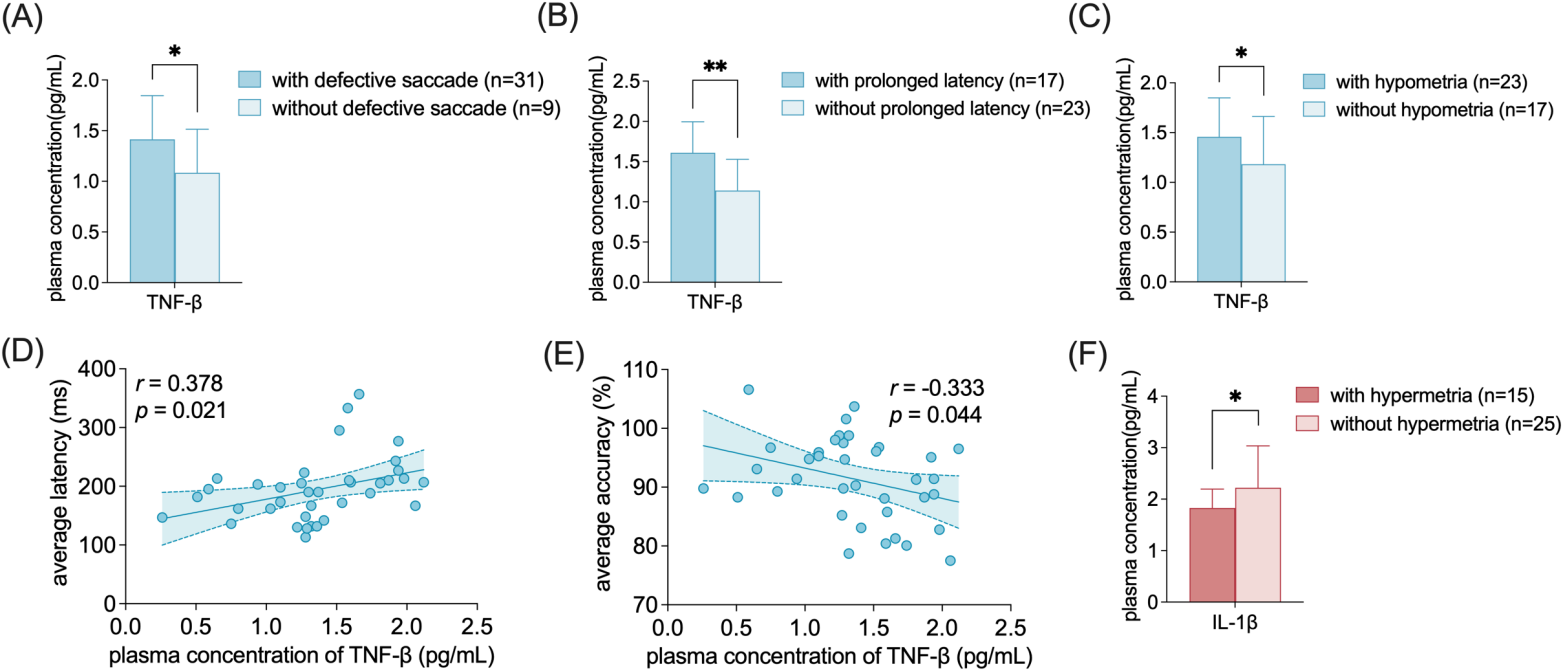
The expression of TNF-β and IL-1β in subgroups of FD patients with saccadic deficits. **(A-C)** Elevated levels of TNF-β in patients with defective saccades, prolonged latency, and hypometria. **(D)** Positive correlation between average saccade latency and the plasma level of TNF-β. **(E)** Negative correlation between average saccade accuracy and the plasma level of TNF-β. **(F)** Decreased level of IL-1β in patients with hypermetria. Data are shown as mean ± SD. TNF, tumor necrosis factor; IL, interleukin. *p<0.05; **p<0.01.

The expression level of TNF-β was significantly higher in patients with defective saccades (1.41 ± 0.43 VS 1.08 ± 0.43, p=0.040), prolonged saccade latency (1.61 ± 0.38 VS 1.14 ± 0.39, p=0.001) and hypometria (1.46 ± 0.39 VS 1.18 ± 0.48, p=0.043) than in patients without those abnormalities (**Figures 2a–c**). The average saccade latency was positively correlated with the level of TNF-β (r=0.378, p=0.021) (**Figure 2d**). The average saccadic accuracy was negatively correlated with the level of TNF-β (r=-0.333, p=0.044) (**Figure 2e**).

The level of IL-1β was significantly lower in patients with hypermetria than patients with normal saccadic accuracy (1.83 ± 0.37 VS 2.22 ± 0.81, p=0.040). (**Figure 2f**).

**Figure 3:**
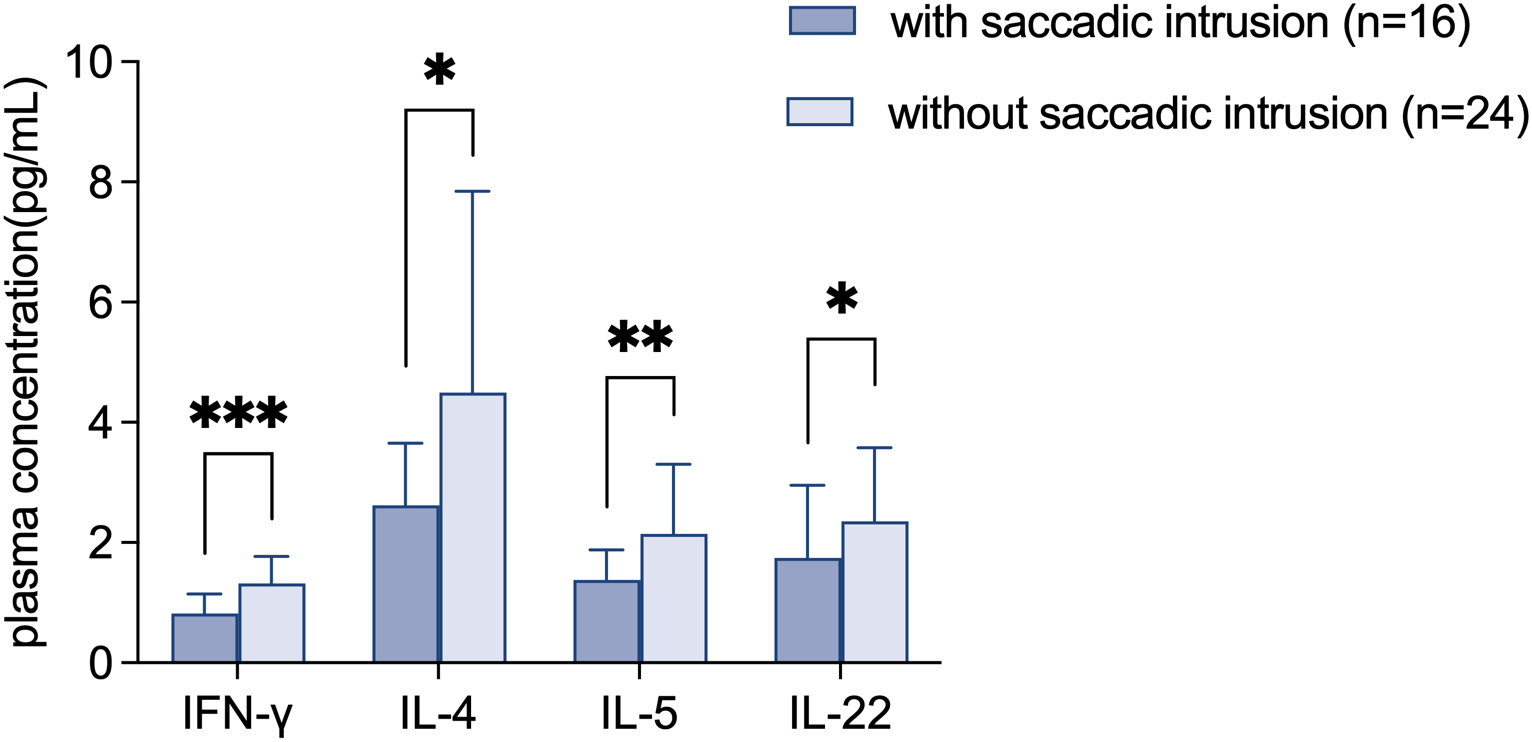
Decreased plasma levels of IFN-γ, IL-4, IL-5 and IL-22 in FD patients with saccadic intrusions. Data are shown as mean ± SD. IFN, interferon; IL, interleukin. *p<0.05; **p<0.01; ***p<0.001.

In patients with saccadic intrusions, the plasma levels of IFN-γ (0.82 ± 0.32 VS 1.32 ± 0.45, p<0.001), IL-4 (2.62 ± 1.03 VS 4.49 ± 3.35, p=0.030), IL-5 (1.38 ± 0.50 VS 2.15 ± 1.16, p=0.008) and IL-22 (1.74 ± 1.21 VS 2.35 ± 1.23, p=0.023) were significantly lower than patients with normal fixation, as shown in **Figure 3**.

#### 3.2.2 Abnormalities in smooth pursuit test

**Figure 4:**
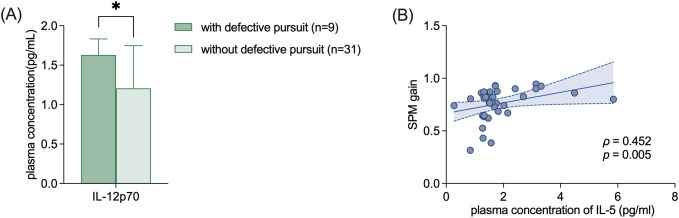
The expression level of ICs in FD patients with and without defective pursuit. **(A)** Elevated plasma level of IL-12p70 in patients with defective pursuit. **(B)** Positive correlation between SPM gain and the plasma level of IL-5. Data of the IL-12p70 and IL-5 concentrations are shown as mean ± SD. Data of SPM gain is shown as median (IQR). IC, inflammatory cytokine; IL, interleukin; SPM, smooth pursuit movement. *p<0.05; **p<0.01.

Nine (22.5%) patients showed saccadic pursuit in SPM test, with 5 (12.5%) of them showed decreased velocity gain. In patients with defective pursuit, as shown in **Figure 4**, the plasma level of IL-12p70 was significantly elevated compared to patients with normal pursuit (1.63 ± 0.20 VS 1.21 ± 0.54, p=0.040) (**Figure 4a**). The SPM gain was positively correlated with the level of IL-5 (ρ=0.452, p=0.005) (**Figure 4B**).

### 3.3 Vestibulo-oculomotor examination changes and the relationship with IC expression

**Figure 5:**
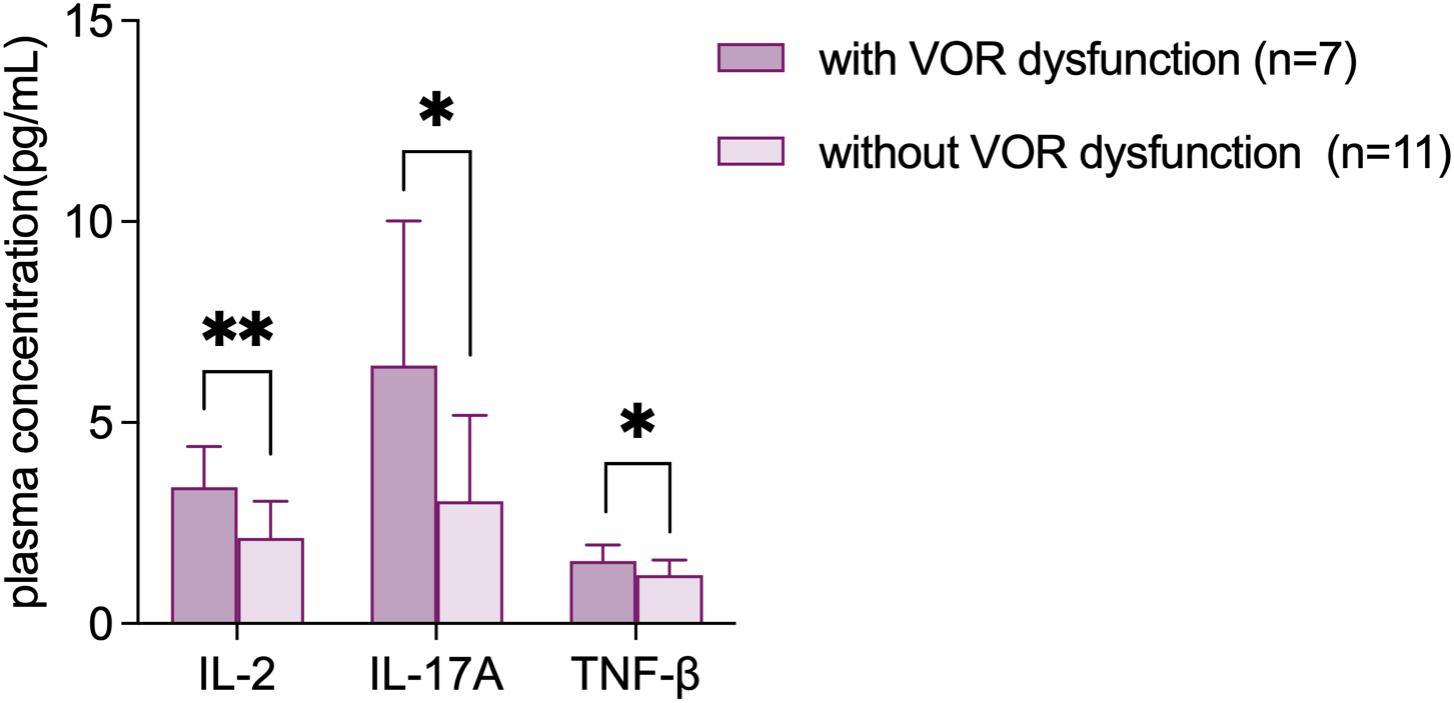
Elevated plasma levels of IL-2, IL-17A and TNF-β in FD patients with VOR dysfunction. Data are shown as mean ± SD. VOR, vestibulo-ocular reflex; IL, interleukin; TNF, tumor necrosis factor. *p<0.05; **p<0.01.

The vestibulo-oculomotor examination was performed in 22 FD patients. Ten (45.4%) of them showed dysfunction of VOR, including 3 patients showed abnormalities in the caloric test only (UW or BW), 3 patients showed decreased gain in the vHIT only, and 4 patients showed abnormalities in both tests. The plasma levels of IL-2 (3.40 ± 1.00 VS 2.13 ± 0.91, p=0.007), IL-17A (6.42 ± 3.59 VS 3.05 ± 2.13, p=0.021) and TNF-β (1.55 ± 0.41 VS 1.21 ± 0.37, p=0.030) in those patients were significantly elevated compared to the patients with normal VOR, which was shown in **Figure 5**.

## 4 Discussion

The current study provides novel evidence supporting the association of vestibular dysfunction in FD with inflammatory mechanisms. The patients with peripheral vestibular damage (impaired VOR) demonstrated elevated IL-2, IL-17A, and TNF-β, while the central vestibular dysfunction was shown to be related with IL-12p70 and TNF-β. Given the role of inflammatory activation in the mechanism of organ damage in FD (10), we can speculate that inflammation may represent a common pathological pathway underlying both systemic organ damage and vestibular dysfunctions in FD.

Sphingolipid deposition plays a critical role in the inflammatory mechanism in FD (10). On the one hand, Gb3 buildup increases lysosomal pH, disrupting autophagy lysosome pathway and generating reactive oxygen species (ROS). On the other hand, Gb3 accumulation in endothelial cells may act as a damage-associated molecular pattern (DAMP) that activates TLR4-mediated NF-κB pathway, promoting leukocyte-derived pro-inflammatory cytokine release and subsequent endothelial injury, which eventually leads to the pathology of heart, kidney and other organs. Our previous work in FD patients demonstrated significant correlations between a variety of plasma ICs and disease burden markers such as MSSI, left ventricular mass index (LVMI), and estimated glomerular filtration rate (eGFR), corroborating the inflammatory basis for the cardiac and renal damage (11). Emerging evidence from animal models and clinical studies implicates the pathological role of Gb3 deposition in cerebral small vessels and the stria vascularis in the inner ear (7, 9). Thus, it can be inferred that Gb3 is also a key link in the inflammation-induced vestibular damage of FD patients.

Autoimmune-mediated pathology typically causes focal damage. The inner ear, being a small anatomical structure containing lymph fluid, is more vulnerable to immune attack (15). Experiments in animal models demonstrate that autoimmune inner ear disease involves the infiltration of TNF-α-expressing cells into endolymphatic sacs (15), while Meniere’s disease is associated with upregulated ICs such as IL-1β, TNF-α, and IL-6, as well as the activation of the NF-κB pathway (16). Even in age-related vestibular impairment, there is an increased expression of proinflammatory genes (17). Although FD similarly involves NF-κB activation, our cohort revealed a distinct IC profile in the subgroup with peripheral vestibulopathy compared to Meniere’s disease. Meanwhile, it is evidenced that the expressions of IL-1β and TNF-β mRNA are upregulated after cochlear ischemia/reperfusion (18), and intraneural injection of TNF-α and TNF-β induces inflammatory cells infiltration and vascular occlusion (19). Our findings of elevated TNF-β levels in FD patients suggest that the process of their peripheral vestibular damage may be more similar to that of ischemia/reperfusion, which may primarily result from the Gb3-induced inflammatory response, leading to the cascade of endothelial injury → vascular constriction or thrombosis → organ ischemia.

Inflammation is also involved in the pathogenesis of cerebrovascular diseases. Elevated inflammatory factors can predict the risk of microbleeds (20). IL-1β, IL-2 and IL-12p70 are increased in the thrombi of patients with acute cerebral infarction (21). TNF-related factors were reported to have causal relationships in cerebral small vessel disease (CSVD) (22). Although our previous study did not establish a correlation between ICs and SVDS in FD (11), the patients with visuo-oculomotor abnormalities (prolonged saccade latency, hypometria, saccadic pursuit) exhibited relatively higher SVDS (9). As the current study showed the associations of TNF-β with saccadic abnormalities, and IL-12p70 with impaired SPM, the central vestibular dysfunction in FD patients may share a similar IC spectrum with CSVD, potentially resulting from Gb3-induced inflammatory cascade in the endothelium of cerebral small vessels.

TNF-β is a pro-inflammation cytokine produced by lymphocytes and crucial for secondary and tertiary lymphoid organ development (23). Previous studies in FD have revealed the contribution of Gb3-related CD27 expression in CD4+ T cells to impaired maturation of memory T cells (24). CD27 is a member of the TNF receptor superfamily (25), the activation of which promotes Th1 cell differentiation (26). Th1 cells secrete TNF-β (27), which in turn activates the NF-κB pathway. Thus TNF-β might be a link in the pathogenesis of FD and widely mediate the multiple-organ damage including the brain and inner ear. Our findings of the close relationship of TNF-β and the vestibular dysfunction support this speculation and suggest a potential value of TNF-β as a biomarker or therapeutic target, pending validation in interventional studies.

Interestingly, although our previous work revealed that IL-5 was elevated in FD patients compared with controls (11), the current data identified a positive correlation with SPM gain, suggesting a somewhat protective effect on central vestibular function. A previous study on multiple sclerosis (MS) demonstrated that the Th2 cell-related factors like IL-4 and IL-5 could limit the activation of Th1 cells, promote the production of anti-inflammatory factors, and have a protective effect on central nervous system demyelination, which might be involved in the therapeutic mechanisms of some medicines (28). Therefore, the mechanism of central vestibular damage in FD may mimic CNS inflammatory demyelinating diseases such as MS, involving the imbalance of Th1/Th2 cell pathway. Moreover, several studies have revealed a potential protective effect of IL-5 in systemic diseases, such as renal fibrosis and diabetic nephropathy (29, 30), implying a biphasic role of this cytokine. It might exert protective effects by restricting Th1 pathway at the early stage of disease, and detrimental effects through inflammation at advanced stages.

Besides IL-5, multiple ICs including IFN-γ, IL-4 and IL-22 were also reduced in our patient subgroup exhibiting saccadic intrusions. However, these findings should be interpreted cautiously. Since our previous work failed to verify a statistical difference in the incidence of saccadic intrusions between FD patients and healthy controls (9), saccadic intrusions were not a FD-specific manifestation. Therefore, the observed reductions of these ICs likely reflect incidental variation rather than protective mechanisms. Saccadic intrusions represent a non-specific fixation disorder originating from dysfunctions of the brainstem/cerebellar gaze stabilization structures. Occasional saccadic intrusions may occur in more than 25% of healthy individuals (31, 32), usually compensated and asymptomatic. While no studies have examined the relationship between saccadic intrusions and inflammatory mechanism in healthy populations, markedly increased saccadic intrusions have been documented in certain autoimmune disorders (33–35). More detailed investigations are needed to establish the role of inflammation in saccadic intrusions, particularly whether these eye movements represent a subclinical autoimmune abnormality.

This study has several important limitations. The modest sample size may have limited the statistical power to detect significant correlations between certain ICs and vestibular function parameters. Considering the exploratory nature of this investigation, adjustments for multiple comparisons were not performed. In future confirmatory research with larger sample size, multiple comparison correction will be necessary to improve statistical robustness. While the current analysis was made within the FD cohort to explore disease−specific associations, future studies comparing IC levels against a disease-control group with other vestibular disorders are needed to more definitively attribute these changes to FD pathology. Utricular and saccular testing should be performed to clarify the influence of otolith organs on vestibular examination results due to utricular-semicircular canal interactions. Besides, enzyme replacement therapy (ERT) is increasingly used in clinical practice. Further studies with longitudinal follow-up, including patients undergoing ERT, should be conducted to assess its impact on vestibular function. This would provide stronger evidence for the causal relationship between Gb3 deposition and vestibular damage.

## 5 Conclusion

Multiple ICs in the plasma of FD patients with central and peripheral vestibular dysfunction are elevated. Notably, TNF-β shows significant associations with both forms of vestibular dysfunctions, demonstrating a potential value as a biomarker or therapeutic target. Inflammatory injury to the endothelium of small vessels, triggered by the Gb3 accumulation, may be a critical mechanism of the vestibular impairment in FD, instructing future validation in larger cohorts or interventional studies.

## Data Availability

The original contributions presented in the study are included in the article/**Supplementary Material**. Further inquiries can be directed to the corresponding author.
