# Supplementary material for: Multiple inflammatory cytokines correlate with the vestibular and oculomotor dysfunction in Fabry disease: a prospective, longitudinal study
Source: Front Immunol. 2026 Apr 17;17:1658002. doi: 10.3389/fimmu.2026.1658002 (PMC13132847; doi:10.3389/fimmu.2026.1658002)
Supplement: Supplementary file 1 [file Table1.docx]

**Multiple Inflammatory Cytokines Correlate with the Vestibular and Oculomotor Dysfunction in Fabry Disease: A Prospective, Longitudinal Study**

**Authors:**

Leng Yinglin^1^**^†^**, Yuan Yujing^1^**^†^**, Zhao Yawen^1^, Wang Xia^1^, Ma Jing^1^, Zhang Xinyu^1^, Huang Jingyi^1^, Hao Hongjun^1,2^, Zhao Guiping^1^, and Zhang Wei^1,3,4#^

1 Department of Neurology, Peking University First Hospital, Beijing, China,

2 Department of Neuroimmunity, Peking University First Hospital, Beijing, China

3 Beijing Key Laboratory of Neurovascular Diseases, Beijing, China

4 Rare disease medical center, Peking University First Hospital, Beijing, China

†These authors contributed equally to this work.

#Correspondence to: Zhang Wei (Email: neurozw@163.com)

Table S1

| Patient ID | *GLA* mutation type | ERT^*^ | Sampling time | Examination time |
| --- | --- | --- | --- | --- |
| P1 | nonsense | Y | Before ERT | Before ERT |
| P2 | missense | Y | After ERT | After ERT |
| P3 | missense | Y | After ERT | After ERT |
| P4 | missense | N |  |  |
| P5 | frameshift | N |  |  |
| P6 | nonsense | Y | Before ERT | After ERT |
| P7 | missense | Y | After ERT | After ERT |
| P8 | frameshift | N |  |  |
| P9 | nonsense | Y | After ERT | Before ERT |
| P10 | nonsense | N |  |  |
| P11 | missense | N |  |  |
| P12 | nonsense | Y | Before ERT | After ERT |
| P13 | missense | Y | Before ERT | After ERT |
| P14 | insertion/deletion | N |  |  |
| P15 | in-frame | Y | Before ERT | After ERT |
| P16 | frameshift | N |  |  |
| P17 | deletion | N |  |  |
| P18 | in-frame | Y | Before ERT | Before ERT |
| P19 | nonsense | Y | Before ERT | Before ERT |
| P20 | missense | N |  |  |
| P21 | missense | N |  |  |
| P22 | missense | N |  |  |
| P23 | missense | N |  |  |
| P24 | missense | N |  |  |
| P25 | nonsense | N |  |  |
| P26 | missense | Y | Before ERT | Before ERT |
| P27 | missense | N |  |  |
| P28 | nonsense | Y | After ERT | After ERT |
| P29 | nonsense | Y | After ERT | After ERT |
| P30 | missense | N |  |  |
| P31 | missense | N |  |  |
| P32 | missense | Y | After ERT | After ERT |
| P33 | missense | N |  |  |
| P34 | missense | Y | Before ERT | Before ERT |
| P35 | missense | N |  |  |
| P36 | splice | N |  |  |
| P37 | nonsense | N |  |  |
| P38 | nonsense | Y | After ERT | After ERT |
| P39 | missense | N |  |  |
| P40 | intron insertion | N |  |  |

ERT: enzyme replacement therapy; *Y: received ERT; N: did not receive ERT
